# Social Partner Effects on Type 2 Diabetes Prevention, Management, and Spillover Health Outcomes: Single-Arm Pre-Post Pilot Intervention

**DOI:** 10.2196/82438

**Published:** 2026-06-08

**Authors:** Natalie B Connell, Sakila Nasrin, Zohra Amin, Nazneen Akhter, Mohammed K Ali, K M Venkat Narayan, Megha Shah

**Affiliations:** 1 Department of Family and Preventive Medicine, Emory University School of Medicine, 100 Woodruff Circle, Atlanta, GA, 30306, United States, 1 1-404-727-4018; 2 Emory University, Emory Global Diabetes Research Center, Woodruff Health Sciences Center, Atlanta, GA, United States

**Keywords:** diabetes, lifestyle intervention, social networks, social partners, household health benefits

## Abstract

**Background:**

South Asian Americans are at high risk of prediabetes and type 2 diabetes mellitus (T2DM). South Asian populations are typically close-knit communities, with support networks that could be leveraged in lifestyle interventions.

**Objective:**

This study was a single-arm, pre-post pilot study to evaluate the feasibility and efficacy of a culturally tailored telehealth intervention for South Asian adults with prediabetes or T2DM and their social partners (trusted household members) who agreed to complete preintervention and postintervention surveys.

**Methods:**

Participants attended 5-hour-long health education sessions delivered in English and Bengali. Participant outcomes included pre-post changes in hemoglobin A1c (HbA_1c_), BMI, blood pressure (BP), self-reported minutes of physical activity, and dietary choices at baseline and at the 6-month follow-up. For social partners, outcomes included pre-post survey changes in physical activity and dietary choices. We used Pearson chi-square tests and paired 2-tailed *t* tests to compare baseline measures with postintervention outcomes.

**Results:**

This pilot study included 54 participants and 106 social partners in Atlanta, Georgia, between March 2021 and November 2023. All participants were Bangladeshi and spoke native Bengali. Social partners were most commonly participants’ children (39/106, 36.8%) or spouses (34/106, 32.1%). The participant baseline HbA_1c_ level was 7.5% (SD 1.48%), which decreased by −0.83% (95% CI 0.42%-1.30%; *P*<.001). Participants also improved systolic BP by −5.8 mm Hg (95% CI 0.196-11.37; *P*=.04) with no change in diastolic BP (−0.451 mm Hg, 95% CI −1.49 to 2.39; *P*=.60) or BMI (−0.642 kg/m^2^, 95% CI −1.87 to 0.59; *P*=.17). Compared with baseline, 39% more participants exercised at least 150 minutes weekly (*P*<.001), but there was no difference in self-reported fruit and vegetable intake. However, the social partners increased fruit and vegetable intake (*P*=.02), decreased soda intake (*P*<.001), and increased daily moderate exercise (*P*=.003).

**Conclusions:**

Including social partners in T2DM prevention and management is feasible and potentially beneficial, but comparative studies are needed to determine the incremental effects of social partners’ participation vs individual-focused lifestyle interventions.

## Introduction

In the United States, ethnic minority groups experience greater rates of prediabetes and type 2 diabetes mellitus (T2DM) when compared with non-Hispanic White individuals [1]. Among ethnic minority groups, South Asian Americans (individuals with ancestry from India, Bangladesh, Sri Lanka, Pakistan, Nepal, Bhutan, and the Maldives) face disproportionately high rates of T2DM [1,2]. South Asian Americans are among the fastest growing ethnic minority groups in the United States, specifically in Georgia, where rates of comorbid cardiovascular disease are especially high [3]. As such, addressing prediabetes and T2DM among South Asians in Georgia is of public health importance.

Despite the known concentration of T2DM affecting South Asians in Georgia, there has been a lack of culturally tailored interventions addressing these risk factors in this population. Barriers to improving the health of South Asians with T2DM include high rates of limited English proficiency, limited access to health insurance and transportation, and lower household income [4,5]. Language-appropriate and culturally adapted community health worker (CHW) interventions have improved the control of T2DM in African American and Latino populations in the United States [6]. Moreover, previous trials have demonstrated that group-based programs that combine diet, activity, and behavior changes reduce complications and provide long-lasting health benefits among people with T2DM [5,6].

Social networks, including family networks, have been identified as potential mediators of the long-term benefits of group-based lifestyle interventions to improve the management of T2DM [7]. Social networks can affect health through social support, influence, and engagement, as well as through more concrete mechanisms such as shared access to resources. Specifically, several recent studies have demonstrated the effects of T2DM diagnosis or management among spouses who are not receiving treatment. Partners of those with recently diagnosed T2DM are at increased risk of developing T2DM and could be considered a high-risk population for screening and prevention [8]. A multicenter randomized controlled trial of weight loss in participants with T2DM demonstrated that behavioral weight loss interventions can be extended to untreated spouses [9].

Social network interventions and spillover effects remain understudied in South Asian American populations, which often have tightly connected families and communities [10,11]. Better Together is a single-arm, pre-post pilot study of an intensive lifestyle telehealth program tailored for South Asian immigrants with prediabetes or T2DM and their household members in Georgia. We aimed to evaluate the feasibility of this intervention and its preliminary effects on participants’ biometric and behavioral outcomes related to T2DM management. We also explored whether the intervention produced spillover behavioral health changes among enrolled social partners and whether including social partners enhanced outcomes for participants. As a pilot study, these findings are preliminary and intended to inform future hypothesis-driven trials.

## Methods

### Study Design

This study analyzed the feasibility and efficacy of the Better Together Atlanta intervention in South Asian Americans with prediabetes and T2DM. We followed the Guidelines for Reporting Nonrandomized Pilot and Feasibility Studies for this report [12].

### Study Recruitment

Recruitment occurred over 3 phases from December 2021 to October 2023, with each phase lasting 6 months. Participant recruitment was community based, leveraging relationships through a community advisory board that consisted of religious and cultural organizations across Georgia. Screened participants were eligible for enrollment if they met the following criteria: (1) South Asian ethnicity; (2) documented hemoglobin A1c (HbA_1c_) of ≥5.7% or fasting blood glucose of >100 mg/dL; (3) aged ≥18 years; and (4) confirmed that at least 1 social partner (trusted household member) was willing to complete preintervention and postintervention surveys. Social partners also had to be aged >18 years and could be any relation to a participant so long as they shared the same household. Ineligibility criteria included the following: (1) pregnant at time of screening; (2) diagnosis of type 1 diabetes or diabetes secondary to other conditions; and (3) inability to perform unsupervised physical activity determined by self-report at screening.

### Ethical Considerations

The study protocol was reviewed and approved by the Emory University Institutional Review Board in September 2020, and all procedures were conducted in accordance with the ethical standards of the Emory Institutional Review Board (Study 00000967). All participants and social partners provided written informed consent prior to study enrollment. The study data were anonymized and deidentified. The intervention was registered at ClinicalTrials.gov (NCT05275231) in November 2021. Consent for publication was obtained via the participant consent form, which was signed prior to study participation.

### Intervention

The 5-session intervention was delivered in Bengali and English by 3 CHWs via video or audio calls, each lasting approximately 60 minutes. Health education session topics included the following: (1) overview of T2DM; (2) nutrition; (3) stress management; (4) healthy weight and physical activity; and (5) T2DM management. All the sessions were tailored to South Asians by discussing religious practices, culturally specific foods, and gender-specific exercises. These sessions were developed for a prior intervention for South Asians with T2DM and prediabetes [11], and further details on the mode of delivery, session content, and cultural tailoring of the intervention have been previously described [13].

Following session 1, participants completed an action plan development form in which participants and CHWs created action plan goals (eg, weight loss and lowering HbA_1c_). CHWs followed up on the action plans via motivational interviewing techniques through 2 one-on-one sessions with intervention participants, either by telephone or in person. Social partners were not required to coattend the CHW-led educational group sessions and instead committed to completing the preintervention and postintervention questionnaires.

### Measures

Feasibility outcomes included recruitment and enrollment metrics (ie, proportion of recruited participants who enroll), proportion of participants who complete all sessions, and baseline and follow-up survey completion.

The primary efficacy health outcome was a change in HbA_1c_ (%) between baseline and the study end point for primary participants. Baseline and end point HbA_1c_ values were recorded either via medical records or via onsite point-of-care testing as part of the study.

Secondary biometric outcomes included preintervention to postintervention change in systolic blood pressure (BP [mm Hg], diastolic BP [mm Hg], weight [kg], and BMI [kg/m^2^]). Height was self-reported by participants, and weight was either measured in person by CHWs, self-reported by participants, or documented via electronic health records. BMI was calculated from weight and height.

Patient-centered lifestyle outcomes included preintervention and postintervention self-reported minutes of physical activity weekly (culturally modified from the Behavioral Risk Factor Surveillance System [BFRSS]) [14], dietary intake (culturally modified from the BFRSS), including daily servings of fruits and vegetables and weekly servings of soda or other sugary beverages, health self-efficacy (adapted from the Bandura self-efficacy scale) [15], and days of poor physical and mental health (BFRSS) [14]. We report measures only with strongly agreed upon recommendations, such as moderate-intensity physical activity for 150 minutes per week [16] and 5 daily combined fruit and vegetable servings [17].

For social partners, behavioral outcome measures included changes in preintervention and postintervention physical activity and dietary intake. This was measured using a survey adapted in which social partners reported behaviors on a scale of 0 to 4 (0=never, 2=about half of the time, and 4=always). We used these behavioral health outcomes (changes in physical activity and diet) to measure “spillover” health effects on social partners, choosing the term “spillover” to specify that the social partners themselves did not have to participate in the 5-session lifestyle intervention but rather were influenced by the changes of the intervention participant.

### Sample Size

Power calculations were performed for HbA_1c_ for primary study participants via a lifestyle program to treat T2DM among South Asian immigrants in New York City to provide appropriate power estimates. They reported a mean −0.2% reduction in HbA_1c_ with an effect size of −0.2 [18]. Using this group as a known population with a baseline HbA_1c_ of 7.8% with an SD of 1.3, a fully powered study to detect a similar difference with an α level of .05 and β level of .20 would require a sample size of more than 300 patients, which was not feasible in this pilot study. Instead, we accepted an α level of .20 and recruited 54 participants, acknowledging that the data from this preliminary study are exploratory and were not intended to confirm the study hypotheses.

### Statistical Analyses

We compared baseline measures to postintervention outcomes for both the study participants and social partners via Pearson chi-square tests for categorical variables (n, %) and paired 2-tailed *t* tests for continuous variables (mean, SD) and change (95% CI). GraphPad (GraphPad Software, Inc) was used for all analyses. Sensitivity analyses were performed for missing data with no change in significant *P* values. We also stratified the social partner data to investigate preintervention and postintervention differences across social partner type (spouse vs child vs other) and gender.

## Results

### Demographic Data

**Figure 1.**
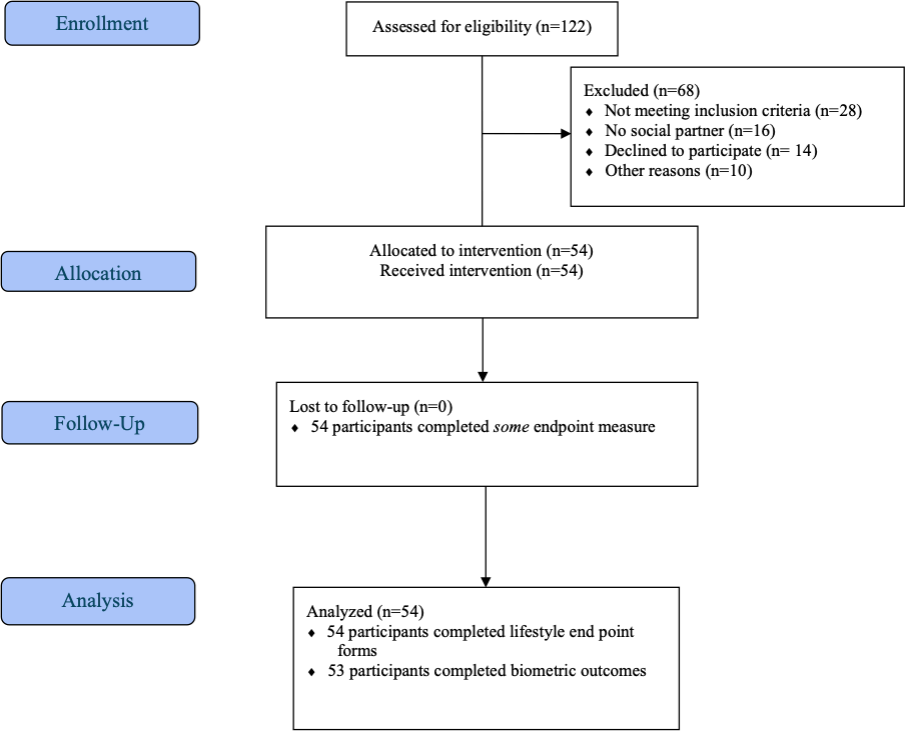
CONSORT (Consolidated Standards of Reporting Trials) diagram of the Better Together study participants.

Patient screening and enrollment information is documented in Figure 1. A total of 54 study participants were recruited for this pilot study, each with 1 to 2 family members or friends for 106 total social partners. At baseline, approximately half (28/54, 51.9%) of the study participants were female, the mean age was 58.2 (SD 10.5) years, all study participants were from Bangladesh, and 63% (34/54) spoke English at a level of “good” or higher (Table 1). Among the social partners who participated in the study, 98 (N=106, 92.5%) were family members, 6 (5.7%) were friends, and the rest (n=2, 1.8%) were identified as “Other.” Most (73/106, 68.9%) of the family members were either spouses or children.

**Table 1.** Demographics of the Better Together participants and social partners.

Characteristics	Values
Participants (n=54)	
Age (years), mean (SD)	58.2 (10.5)
Living in the United States (years), median (IQR)	10.0, (5.0-22.0)
Sex, n (%)	
Male	26 (48.1)
Female	28 (51.9)
Birth country, n (%)	
Bangladesh	54 (100)
Native language	
Bengali	53 (98.1)
Other or skipped	1 (1.9)
English fluency	
Very good	13 (24.1)
Good	21 (38.9)
Not good	16 (29.6)
Not good at all	4 (7.4)
Language spoken at home	
Mostly English	0 (0)
Mix of English and native language	10 (18.5)
Mostly native language	43 (79.6)
Other or skipped	1 (1.9)
Education	
Some high school	8 (14.8)
High school graduate	5 (9.3)
Some college	5 (9.3)
College graduate	33 (61.1)
Employment status	
Employed for wages	24 (44.4)
Self-employed	1 (1.9)
Homemaker	11 (20.4)
Student	1 (1.9)
Retired	13 (24.1)
Unemployed	4 (7.4)
Marital status	
Married	45 (83.3)
Divorced	1 (1.9)
Widowed	7 (13)
Social partners (n=106)	
Relationship	
Family member	98 (92.5)
Close friend	6 (5.7)
Other or skipped	2 (1.9)
Family relation	
Spouse	34 (32.1)
Child	39 (36.8)
Grandchild	1 (0.9)
Sibling	1 (0.9)
Parent	2 (1.9)
Grandparent	1 (0.9)
Niece or nephew	5 (4.7)
Other or skipped	22 (21.7)

### Feasibility Outcomes

All 54 participants completed intake and end point forms. Most participants (39/54, 72.2%) completed all 5 intervention sessions, and all participants completed action plans and both one-on-one sessions, either via video calls (n=53, 98%) or a telephone (n=1, 2%). Most of the 5 educational CHW-led sessions were group sessions (30/35, 86%). Only 6 (5.7%) social partners attended the CHW-led sessions throughout the intervention. Of the 106 social partners who completed preintervention surveys, 104 (98.1%) completed postintervention surveys.

### Preliminary Participant Outcomes: HbA_1c_ and Biometric

The baseline HbA_1c_ for the study participants was 7.5% (SD 1.49%; Table 2), and by the end of the 5-session intervention, the average HbA_1c_ decreased by −0.83% (95% CI 0.42%-1.3%; *P*<.001) to 6.7% (SD 0.81%). On average, study participants experienced a −1.33 kg reduction (95% CI −3.24 to 0.58; *P*=.17) throughout the course of the intervention, with no significant accompanying change in BMI (−0.642 kg/m^2^, 95% CI −1.87 to 0.59; *P*=.17). Mean systolic BP at baseline was 129.7 (SD 19.40) mm Hg, with an average reduction of −5.78 mm Hg (95% CI 0.196-11.37; *P*=.04) after the intervention. Diastolic BP at baseline was 76.3 (SD 7.65) mm Hg and did not change after the intervention (−0.451 mm Hg, 95% CI −1.49 to 2.39; *P*=.64).

**Table 2.** Changes in clinical measurements of study participants from baseline to after the intervention.

	Baseline, mean (SD)	After the intervention, mean (SD)	Change (95% CI)	*P* value
HbA_1c_^a^ (%)	7.5 (1.49)^b^	6.7 (0.81)^c^	−0.83 (0.42 to 1.3)	.002^d^
Body weight (kg)	70.3 (11.8)^e^	69.3 (11.6)^b^	–1.33 (–3.24 to 0.58)	.17
BMI (kg/m^2^)	26.56 (3.70)^e^	25.92 (4.94)^b^	−0.624 (−1.87 to 0.589)	<.001^d^
Systolic BP^f^ (mm Hg)	129.7(19.40)^b^	123.9(13.95)^c^	−5.784 (0.196 to 11.37)	.04^g^
Diastolic BP (mm Hg)	76.3 (7.65)^b^	76.0 (7.57)^c^	−0.451 (−1.490 to 2.392)	.64

a HbA_1c_: hemoglobin A1c.

b n=53.

c n=51.

d *P*<.001.

e n=54.

f BP: blood pressure.

g *P*<.05.

### Preliminary Participant Outcomes: Lifestyle Health Behaviors

Compared with before the intervention, 39% more participants exercised at least 150 minutes weekly after the intervention (*P*<.001; Table 3), but there was no difference in self-reported daily fruit and vegetable servings (−0.085 combined servings; *P*=.66). A total of 55.5% (30/54) of the participants reported drinking no soda before the intervention, which increased to 70.4% (38/54) after the intervention (*P*=.12). When asked about the consumption of sugary beverages other than soda, 64.8% (35/54) of participants reported drinking none after the intervention compared with 44.4% (24/54) at baseline (*P*=.03).

**Table 3.** Changes in lifestyle behaviors of study participants from baseline to after the intervention.

	Baseline	After the intervention	Change (95% CI)	*P* value
Weekly exercise ≥150 min/week, n (%)^a^	18 (33.3.0)	40 (74.0)	—^b^	<.001^c^
Drinks no soda, n (%)^a^	30 (55.5)	38 (70.4)	—	.12
Drinks no other sugary beverages, n (%)	24 (44.4)	35 (64.8)	—	.03^d^
Daily combined fruit and vegetable servings, mean (SD)	2.140 (1.30)	2.055 (1.316)	−0.853 (−0.47 to 0.31)	.66

a *df*=1.

b Not applicable.

c *P*<.001.

d *P*<.05.

### Preliminary Social Partner Outcomes: Lifestyle Health Behaviors

Social partners reported eating significantly more daily fruit and vegetable servings after the intervention (*P*=.02; Table 4). Of the 106 social partners, 26 (24.5%) set a daily calorie goal at least most of the time before the intervention; this percentage increased to 34% (n=36) of social partners after the intervention (*P*=.007). After the intervention, 23.6% more social partners limited their intake of soda at least most of the time (*P*<.001). Significantly more social partners also reported increasing their moderate to high intensity exercise after the intervention (*P*=.003).

**Table 4.** Changes in lifestyle behaviors of social partners from baseline to after the intervention (N=106)

	Baseline responses			Responses after the intervention			*P* value
	n (%)	Mean (SD)	Median (IQR)	n (%)	Mean (SD)	Median (IQR)	
Fruits and vegetable intake							
I had several servings of fruits and/or vegetables each day^a^		2.15 (1.17)	2 (1-3)		2.66 (1.11)	3 (2-3)	—^b^
0^c^	10 (9.4)			5 (4.7)			
1^d^	22 (20.8)			11 (10.4)			
2^e^	29 (27.3)			23 (21.7)			
3^f^	32 (30.2)			39 (36.8)			
4^g^	13 (12.3)			25 (23.6			
Blank	0 (0.0)			3 (2.8)			
Calorie goal							
I set a daily calorie goal for myself^a^		1.24 (1.38)	1 (0-2)		1.65 (1.35)	1 (1-3)	.007^h^
0	49 (46.2)			26 (24.5)			
1	17 (16.0)			31 (29.3)			
2	14 (13.2)			10 (9.4)			
3	18 (16.9)			27 (24.6)			
4	8 (7.6)			10 (9.4)			
Blank	0 (0.0)			2 (1.9)			
Beverages							
I limited my intake of regular soda^a^		2.19 (1.40)	3 (1-3)		3.04 (1.16)	3 (3-4)	<.001^h^
0	14 (13.2)			5 (4.7)			
1	30 (28.3)			9 (8.5)			
2	8 (7.5)			10 (9.4)			
3	30 (28.3)			32 (30.2)			
4	24 (22.6)			47 (44.3)			
Blank	0 (0.0)			3 (2.8)			
Exercise							
I engaged in moderate-intensity exercise like brisk walking or something like brisk walking for at least 30 minutes a day^a^		1.48 (1.42)	1 (0-3)		2.18 (1.36)	2 (1-3)	.003^h^
0	36 (34.0)			17 (16.0)			
1	26 (24.5)			18 (17.0)			
2	15 (14.2)			17 (16.0)			
3	15 (14.2)			33 (31.1)			
4	14 (13.2)			19 (17.9)			
Blank	0 (0.0)			2 (1.9)			

a *df*=5.

b Not applicable.

c 0=never.

d 1=occasionally.

e 2=half of the time.

f 3=most of the time.

g 4=always.

h *P*<.05.

When stratifying by social partner type (spouse vs child vs other), the spouses of participants reported an increase in engagement in moderate to high intensity exercise (*P*=.02) and more frequently limited their regular soda intake (*P*<.001) after the intervention. Children of participants demonstrated no significant changes before and after the intervention. Other family members (ie, niece, nephew, and son-in-law) and close friends reported increased use of daily calorie goals (*P*=.02) after the intervention. When stratifying social partners by sex, female social partners reported increased use of daily caloric goals (*P*=.02) and limited soda intake (*P*<.001) after the intervention, while male social partners reported increased fruit and vegetable intake (*P*=.046) and increased moderate to high intensity exercise (*P*=.02) after the intervention.

## Discussion

### Principal Findings

In this pre-post evaluation of a culturally adapted lifestyle intervention for South Asian Americans with prediabetes and T2DM, the intervention was feasible and demonstrated preliminary efficacy in improving HbA_1c_ and lifestyle health behaviors. Nearly half (25/54, 46%) of the participants experienced clinically significant reductions in HbA_1c_ (≥0.5%) from before the intervention to after the intervention. Participants also reported positive changes in moderate-intensity physical activity and dietary changes for T2DM management, demonstrating the value of a lifestyle intervention that specifically uses established social networks. Additionally, this pilot intervention demonstrated “spillover” behavioral health effects on social partners, as evidenced by improved dietary choices and increased engagement in moderate-intensity exercise.

This telehealth lifestyle intervention was met with high participant engagement and retention. Only 1 participant attended fewer than 4 CHW-led sessions, and all participants attended one-on-one sessions with the CHWs, indicating that this pilot lifestyle intervention is appropriate for future randomization with larger sample sizes. Requiring surveys of social partners also was feasible, as 98% (104/106) of social partners completed both preintervention and postintervention surveys. Feasibility of including social partners in intervention sessions was not studied. Notably, the improvements in participant HbA_1c_ as well as participant and social partner diet and exercise changes offer an efficacy signal for robustly powering future trials.

Our findings on participant outcomes are consistent with those of other CHW-led lifestyle interventions for minority patients with T2DM. In a similar culturally tailored telehealth intervention in Atlanta (Diabetes Research, Education, and Action for Minorities Atlanta; DREAM), South Asian Americans with comorbid T2DM and hypertension participated in a 5-session CHW intervention and reported increased weight loss, increased fruit intake, decreased sweetened beverage intake, and increased physical activity compared with controls not participating in CHW-led sessions [11]. The data from Better Together demonstrate comparable weight loss and similar changes in lifestyle behaviors. As our intervention focused specifically on patients with T2DM or prediabetes and not patients with comorbid hypertension, we observed a lesser improvement in BP outcomes but a greater reduction in mean HbA_1c_. Specifically, participants in our pilot study observed an average HbA_1c_ reduction of −0.83%, while the DREAM study participants demonstrated an HbA_1c_ reduction of −0.4%. Interestingly, the DREAM study included a control group who underwent treatment as usual that observed a mean average HbA_1c_ reduction of only −0.2%, highlighting the potential efficacy of a culturally tailored lifestyle intervention and social partner partnership in reducing HbA_1c_ over treatment as usual for South Asian Americans. Another similar lifestyle program to treat T2DM among South Asian immigrants in New York City demonstrated a mean HbA_1c_ reduction of −0.2% in the intervention group and 0.0% in the control group [18]. While conclusions are limited in a pilot study and in the absence of further statistical analyses, one potential mediator of the increased HbA_1c_ reduction seen in our study population could be the novel inclusion of social partners. This warrants further investigation in a fully powered study with appropriate controls.

Spillover effects were evident: social partners achieved several key improvements in health behaviors, namely, increased fruit and vegetable intake, increased engagement in high-intensity exercise, and decreased soda intake. Within the literature on family- and couple-based interventions, spousal health effects have been well studied, and there is compelling evidence that the health of one partner can affect the health of the other [19]. Our pilot study adds to this evidence by explicitly demonstrating mutually beneficial health effects on both study participants and household members. In total, 32% (34/106) of the social partners in this intervention identified as participant spouses, and we observed more significant changes in this group (increased exercise and decreased soda intake) than other types of social partners. Other social partners were commonly children of study participants, whose reciprocal health effects are less well established [7,19]. Similarly, in our study, children of participants alone did not demonstrate any significant changes before and after the intervention.

Importantly, most social partners did not attend CHW-led educational sessions. Other family or couple-based interventions demonstrating reductions in HbA_1c_ have relied on coparticipation and dyadic goal setting as mechanisms of improvement in diabetes management [20]. However, this pilot study contributes to the nascent body of work pointing to social support, communal coping, and positive perceptions of disease and disease management as potential mediators of health behavior changes [21].

Our study has several limitations. The small sample size and the absence of a control group limit the inferences we can draw about the efficacy and potential benefits of the Better Together intervention. Similarly, we are unable to separate the effects of lifestyle interventions (educational and one-on-one sessions) from the effects of social partner participation. While we tracked social partner attendance at CHW-led sessions, we did not survey participants or social partners about other supportive behaviors (ie, exercising or meal planning together and perceived benefits of social partner inclusion), and as such, inferences regarding the exact mechanisms of social influence are limited. However, we are undertaking a qualitative study to gain more insight into intervention mechanisms of change, acceptability, and satisfaction.

### Conclusions

Our pilot study adds to a growing body of literature supporting culturally tailored lifestyle interventions for T2DM prevention and management. Our findings also support nascent evidence for considering social networks in the prevention and management of T2DM, with bidirectional health benefits for patients and household members. Powered randomized trials are needed to determine the effects of social partner participation vs standard lifestyle interventions. More broadly, these findings suggest that integrating social partners into culturally tailored lifestyle interventions may amplify behavior change beyond the individual participant and into the household. If confirmed in larger, controlled trials, this approach could inform scalable strategies for T2DM prevention and management in South Asian and other underserved populations.

## Abbreviations

BFRSS: Behavioral Risk Factor Surveillance System
BP: blood pressure
CHW: community health worker
HbA_1c_: hemoglobin A1c
T2DM: type 2 diabetes mellitus
